# Anatomical Quantitative Volumetric Evaluation of Liver Segments in Hepatocellular Carcinoma Patients Treated with Selective Internal Radiation Therapy: Key Parameters Influencing Untreated Liver Hypertrophy

**DOI:** 10.3390/cancers16030586

**Published:** 2024-01-30

**Authors:** Raphaël Girardet, Jean-François Knebel, Clarisse Dromain, Naik Vietti Violi, Georgia Tsoumakidou, Nicolas Villard, Alban Denys, Nermin Halkic, Nicolas Demartines, Kosuke Kobayashi, Antonia Digklia, Niklaus Schaefer, John O. Prior, Sarah Boughdad, Rafael Duran

**Affiliations:** 1 Department of Radiology and Interventional Radiology, Lausanne University Hospital and Lausanne University, 1011 Lausanne, Switzerland; girardet.raphael@gmail.com (R.G.); jean.francois.knebel@gmail.com (J.-F.K.); clarisse.dromain@chuv.ch (C.D.); naik.vietti-violi@chuv.ch (N.V.V.); georgia.tsoumakidou@chuv.ch (G.T.); nicolas.villard@chuv.ch (N.V.); alban.denys@chuv.ch (A.D.); 2 Department of Visceral Surgery, Lausanne University Hospital and Lausanne University, 1011 Lausanne, Switzerland; nermin.halkic@chuv.ch (N.H.); demartines@chuv.ch (N.D.); kosuke.kobayashi@jfcr.or.jp (K.K.); 3 Division of Hepatobiliary and Pancreatic Surgery, Cancer Institute Hospital, Japanese Foundation for Cancer Research, Tokyo 135-8550, Japan; 4 Department of Medical Oncology, Lausanne University Hospital and Lausanne University, 1011 Lausanne, Switzerland; antonia.digklia@chuv.ch; 5 Department of Nuclear Medicine and Molecular Imaging, Lausanne University Hospital and Lausanne University, 1011 Lausanne, Switzerland; niklaus.schaefer@chuv.ch (N.S.); john.prior@chuv.ch (J.O.P.); sarah.boughdad@chuv.ch (S.B.)

**Keywords:** hepatocellular carcinoma, selective internal radiation therapy, radioembolization, future liver remnant, liver volume, liver hypertrophy

## Abstract

**Simple Summary:**

Selective internal radiation therapy (SIRT) is widely used for hepatocellular carcinoma (HCC) treatment. Following SIRT, complex morphological changes in the liver occur, with hypertrophy of the untreated liver and atrophy of the treated liver. However, the factors affecting these morphological changes are still unclear. This study aimed to investigate liver volume changes after SIRT for HCC with different levels of treatment selectivity and to evaluate the parameters affecting these changes using a segmentation-based 3D software relying on liver vascular anatomy. Our results, based on a cohort of 88 HCC patients treated with SIRT, showed that younger patients with smaller spleen volume, higher administered ^90^Y activity, and larger amount of treated liver had a higher degree of untreated liver hypertrophy. When SIRT is used in potential surgical candidates, these parameters should be considered to improve patient selection.

**Abstract:**

**Background**: Factors affecting morphological changes in the liver following selective internal radiation therapy (SIRT) are unclear, and the available literature focuses on non-anatomical volumetric assessment techniques in a lobar treatment setting. This study aimed to investigate quantitative changes in the liver post-SIRT using an anatomical volumetric approach in hepatocellular carcinoma (HCC) patients with different levels of treatment selectivity and evaluate the parameters affecting those changes. This retrospective, single-institution, IRB-approved study included 88 HCC patients. Whole liver, liver segments, tumor burden, and spleen volumes were quantified on MRI at baseline and 3/6/12 months post-SIRT using a segmentation-based 3D software relying on liver vascular anatomy. Treatment characteristics, longitudinal clinical/laboratory, and imaging data were analyzed. The Student’s *t*-test and Wilcoxon test evaluated volumetric parameters evolution. Spearman correlation was used to assess the association between variables. Uni/multivariate analyses investigated factors influencing untreated liver volume (uLV) increase. **Results**: Most patients were cirrhotic (92%) men (86%) with Child–Pugh A (84%). Absolute and relative uLV kept increasing at 3/6/12 months post-SIRT vs. baseline (all, *p* ≤ 0.005) and was maximal during the first 6 months. Absolute uLV increase was greater in Child–Pugh A5/A6 vs. ≥B7 at 3 months (A5, *p* = 0.004; A6, *p* = 0.007) and 6 months (A5, *p* = 0.072; A6, *p* = 0.031) vs. baseline. When the Child–Pugh class worsened at 3 or 6 months post-SIRT, uLV did not change significantly, whereas it increased at 3/6/12 months vs. baseline (all *p* ≤ 0.015) when liver function remained stable. The Child–Pugh score was inversely correlated with absolute and relative uLV increase at 3 months (rho = −0.21, *p* = 0.047; rho = −0.229, *p* = 0.048). In multivariate analysis, uLV increase was influenced at 3 months by younger age (*p* = 0.013), administered ^90^Y activity (*p* = 0.003), and baseline spleen volume (*p* = 0.023). At 6 months, uLV increase was impacted by younger age (*p* = 0.006), whereas treatment with glass microspheres (vs. resin) demonstrated a clear trend towards better hypertrophy (f = 3.833, *p* = 0.058). The amount (percentage) of treated liver strongly impacted the relative uLV increase at 3/6/12 months (all f ≥ 8.407, *p* ≤ 0.01). **Conclusion**: Liver function (preserved baseline and stable post-SIRT) favored uLV hypertrophy. Younger patients, smaller baseline spleen volume, higher administered ^90^Y activity, and a larger amount of treated liver were associated with a higher degree of untreated liver hypertrophy. These factors should be considered in surgical candidates undergoing neoadjuvant SIRT.

## 1. Introduction

Following selective internal radiation therapy (SIRT) of hepatocellular carcinoma (HCC) patients, morphologic liver changes are observed. These observations are made after lobar treatment (mainly unilobar) [1,2,3,4,5,6,7,8,9]. Liver remodeling takes place over several months [2,3,5]. Time-dependent atrophy of the treated liver and hypertrophy of the untreated liver (range 26–47% at 44 days to 9 months [10]) are expected after SIRT in a majority of patients, although modest (<10%) or no increase is also reported [2].

Factors inducing those changes are not well known. Recent evidence showed that non-tumoral perfused absorbed dose, preserved liver function before SIRT, age, low tumor burden, and signs of portal hypertension might influence contralateral untreated liver hypertrophy in the setting of unilobar treatment [6,7].

For patients with underlying chronic liver disease eligible for liver surgery, when the future liver remnant (FLR) is <40%, SIRT can be used as a bridge-to-resection. Although less efficient than portal vein embolization in hypertrophying the FLR, the main advantage of SIRT is synchronous tumor treatment [11]. This dual property makes SIRT an appealing technique for the bridge-to-resection scenario. In this context, there is a clinical need to better understand hepatic volumetric changes post-SIRT and the factors influencing those changes. However, most published literature reported pooled data on primary and secondary liver cancer patients [1,3,4,8,9,12], some on liver metastases [13,14,15], with few studies assessing HCC patients alone [2,5,6,7,16]. Moreover, most observations were made after lobar treatment (mostly unilobar) [1,2,3,4,5,6,7,8,13], with few reports focusing on whole-liver or sequential bilobar treatment [12,15], which may not adequately reflect clinical practice as SIRT is increasingly performed in a selective manner. Furthermore, reported volumetric analyses were heterogeneous and non-anatomical, i.e., not based on the liver vascular anatomy. However, liver resection mostly relies on a liver-segment-based, anatomical approach. Therefore, previous work may not reflect the real liver volume evolution after SIRT. Methods considering liver anatomical complexity and vascular variability are required, especially for potential surgical candidates. 

We aimed to investigate three-dimensional (3D) quantitative changes in the liver following SIRT using an anatomical, surgically validated, volumetric approach in HCC patients and to evaluate the parameters affecting those changes.

## 2. Materials and Methods

This retrospective monocentric study of a prospectively collected patient cohort was approved by the Institutional Review Board (CER-VD #2017−02304). Informed consent was waived.

### 2.1. Study Design

HCC patients (*n* = 112) were treated with SIRT (2011–2018). All patients were reviewed in a multidisciplinary liver tumor board and provided informed consent for SIRT.

Inclusion criteria: (1) HCC proven by means of biopsy and/or imaging [17]; (2) HCC treated with SIRT; (3) MRI before and at least one follow-up at 3/6 or 12 months. To reflect clinical practice, our cohort was composed of miscellaneous SIRT types, including whole liver, because we wanted to assess the regenerative ability of the liver in different scenarios.

**Figure 1:**
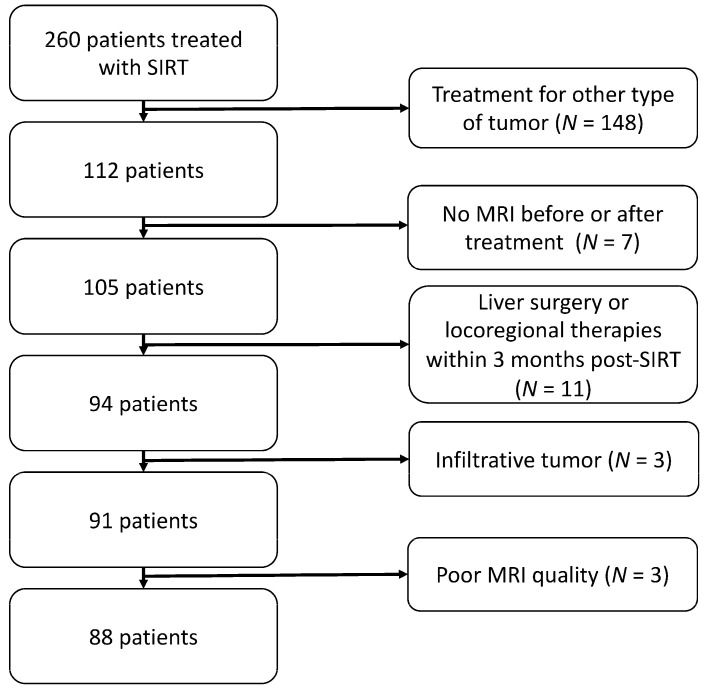
Patients’ inclusion flowchart.

Exclusion criteria: (1) Liver surgery or locoregional therapies before SIRT; (2) infiltrative-type HCC; (3) MRI with artefacts. Of note, MRIs in patients who had liver-directed therapies or surgery/transplantation following SIRT were not considered in the analysis. The final study population included 88 patients (Figure 1).

### 2.2. Data Collection

Clinical and laboratory parameters were collected before and after treatment using health records.

### 2.3. Treatment

Simulation angiography was performed for therapy planning using the partition model, followed by SIRT, as previously reported (Text S1) [18,19,20,21,22].

### 2.4. MRI 

Liver MRI was performed using a standard protocol (Text S2). 

### 2.5. Image Analysis

#### 2.5.1. Three-Dimensional Quantitative Analysis

Detailed volumetric analysis was performed for each patient at each time point (pre-treatment, 3, 6, and 12 months post-treatment) using software (Synapse 3D v5.5, Fujifilm, Tokyo, Japan) for a total of 246 MRIs. This software was chosen because volume analysis is based on vascular anatomy to obtain the liver segments, similar to liver surgery. Its accuracy was validated through radiologic–pathologic correlations, and it is currently widely used in liver surgery planning [23,24].

Collected data included whole liver, right and left liver/lobe, individual segments (I–VIII according to Couinaud’s segmentation), spleen, and tumor burden volumes. Liver volumes are presented as absolute (mL) and relative volumes (%, referred to as the whole liver volume).

**Figure 2:**
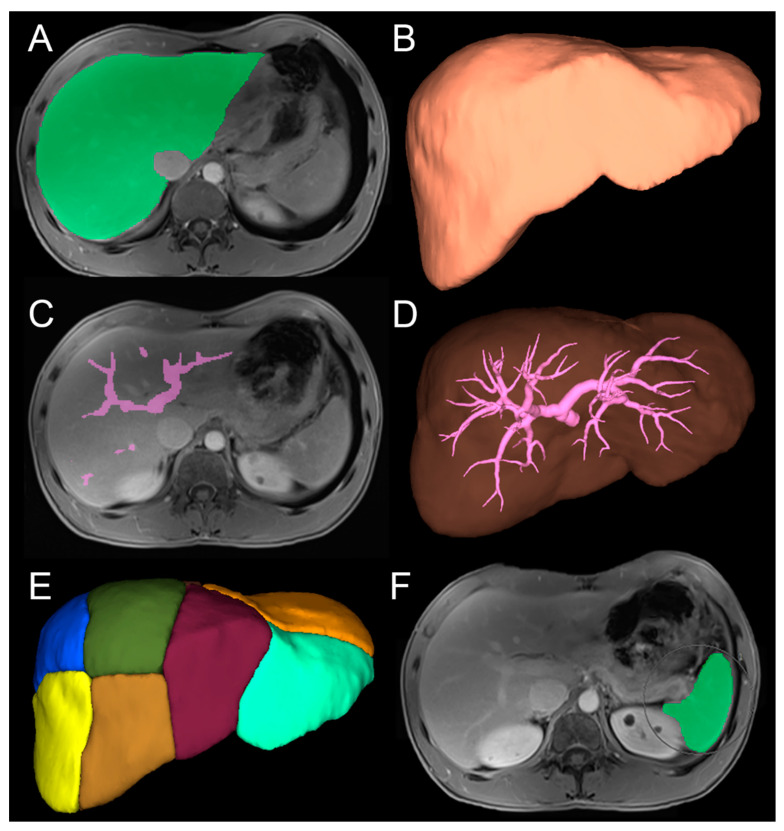
Anatomical 3D quantitative assessment of liver segments, tumor burden, and spleen volumes. (**A**) Semi-automatic volumetric drawing of liver mask (portal venous phase of MRI). (**B**) Total liver volume. (**C**) Manual drawing of each segmental portal vein (portal venous phase). (**D**) Portal system in 3D. (**E**) Final result with all segments. (**F**) Semi-automatic contouring of tumors (arterial phase) and spleen (portal phase) to obtain respective volumes.

Detailed steps of liver volume assessment are shown in Figure 2. To achieve liver volume assessment, the software performs a semi-automatic 3D liver segmentation, allowing total liver volume calculation. Segmental portal venous branches are selected manually. Based on the branch allocation, the software performs automatic calculation of the segment volumes. Hepatic veins are also displayed. Finally, tumors and spleen volumes are also obtained by semi-automatic contouring. 

#### 2.5.2. Two-Dimensional Qualitative Analysis

Presence, type (partial vs. complete), and extent (trunk, lobar, sub-/segmental) of portal vein thrombosis (PVT) were assessed by 2 radiologists (RG and RD). Any discrepancy was resolved by consensus.

### 2.6. Statistical Analysis

Data were summarized using descriptive statistics. Paired Student’s *t*-test and Wilcoxon signed-rank tests were used to evaluate volumetric parameters evolution, as appropriate. The Spearman correlation coefficient assessed the association between investigated variables as appropriate. Linear regression analysis assessed the influence of investigated variables on volume evolution. A *p*-value < 0.05 was considered significant. Statistical analysis was performed using Anaconda (Python Language Reference, version 2.7), the Python module lifelines, and Rpy2 to link Python with R 3.1.3 (R Foundation). 

## 3. Results

Baseline characteristics are summarized in Table 1. 

### 3.1. Three-Dimensional Quantitative Analysis of Individual Liver Segments

Overall, a decrease in absolute and relative treated segment volumes was still observed at 12 months post-SIRT, except for segment I. This reached statistical significance at all time points for right-sided segments most frequently treated in our cohort (Table S1). Similarly, the compensatory hypertrophy was better captured by absolute untreated liver segment volume increase in the left-sided segments (II–III–IV) and was still observed at 12 months post-SIRT (Table S2).

### 3.2. Three-Dimensional Quantitative Analysis of Liver, Spleen, and Tumor

**Figure 3:**
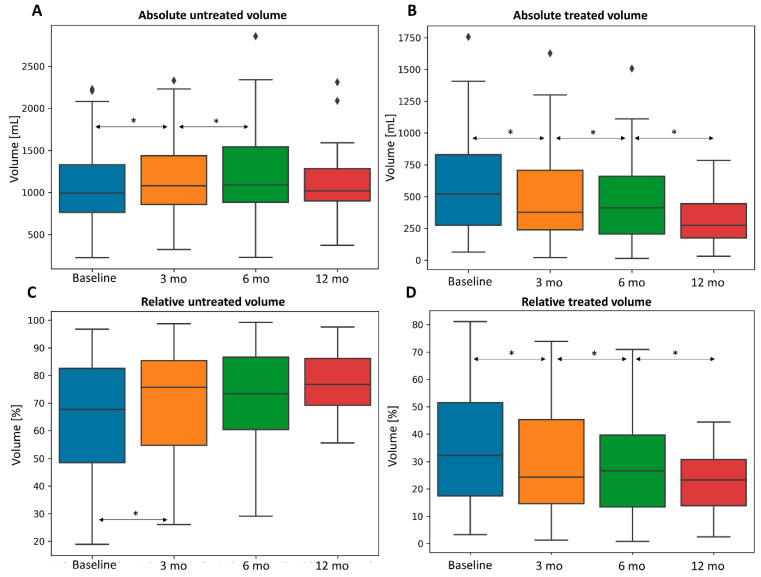
Evolution of liver volumes at 3, 6, and 12 months. Box plot evolution of absolute untreated (**A**) and treated (**B**) liver volumes and relative untreated (**C**) and treated (**D**) liver volumes. * *p* < 0.05.

Volume evolution according to the treated region is presented in Table 2 and Table S3 and overall volume evolution is presented in Figure 3.

In the whole cohort, treated liver volume significantly decreased over time at all time points (all *p* < 0.001; Table 2 and Table S3). Untreated liver volume (uLV) significantly increased at 3, 6, and 12 months (absolute: all *p* ≤ 0.005—Table 2; relative: all *p* < 0.001—Table S3) post-SIRT vs. baseline, although the increase was more pronounced during the first 6 months following therapy.

The evolution of liver volume differs depending on treatment selectivity. After whole liver treatment, absolute volumes decreased over time but only statistically significantly from baseline to 3 months (1530 vs. 1474 mL, *p* = 0.037, respectively, Table 2). After right/left liver/lobe treatment, absolute and relative uLV significantly increased at all time points, even between 6 and 12 months post-SIRT (all *p* ≤ 0.038), whereas treated liver volumes significantly decreased over time (all time points for absolute and relative values, *p* < 0.001; Table 2 and Supplementary Table S3). For more selective treatments, median absolute uLV increased from baseline (1270 mL (224–2230)) to 3 months (1346 mL (379–2329)) and 6 months (1366 mL (390–2857)), although not significantly. However, relative uLV did significantly increase at 3 and 6 months when compared to baseline (*p* < 0.001 and *p* = 0.025, respectively; Table 2 and Table S3).

Treatment selectivity impacted spleen volume increase (Table 2). Hepatic/lobar SIRTs induced significant spleen volume increase at 3, 6, and 12 months vs. baseline (all, *p* ≤ 0.02). A marked increase in spleen volumes was also observed over time after whole liver SIRT, although not significant in this small treatment subgroup. With more selective therapy, spleen volume did not change significantly or even decrease at 3 months vs. baseline (median, 376 vs. 391 mL, respectively, *p* = 0.023; Table 2).

Absolute treated tumor volume did not change significantly early post-SIRT (at 3 months) in all patients and treatment subgroups (except whole liver treatment), but then significantly decreased in all patients over time, between 3 and 6 months and 6 and 12 months (all *p* ≤ 0.03, Table 2).

### 3.3. Factors Influencing Volumetric Changes

Uni/multivariate analyses of variables associated with untreated liver hypertrophy are shown in Table 3 and Table 4.

#### 3.3.1. Clinical Parameters 

Sex, ECOG, and cirrhosis did not influence uLV evolution.

However, age impaired absolute untreated liver hypertrophy at 3 and 6 months in univariate (rho = −0.35, *p* =< 0.001 and rho = −0.27, *p* = 0.043) and multivariate analysis (*p* = 0.013 and *p* = 0.006) (Table 3). The influence found between age and relative untreated liver hypertrophy on univariate analysis at 3 and 6 months (rho = −0.253, *p* = 0.028 and rho = −0.283, *p* = 0.069) did not remain significant after multivariate analysis (Table 4).

#### 3.3.2. Liver Function

Baseline total bilirubin inversely correlated (rho = −0.279, *p* = 0.009), whereas albumin, platelets, and prothrombin time (PT) positively correlated (rho = 0.224, *p* = 0.039; rho = 0.212, *p* = 0.048, rho = 0.282, *p* = 0.008, respectively), with absolute uLV increase at 3 months, but not later on. Similar results were obtained for relative uLV increase. No correlation was found between baseline aspartate/alanine transaminases, alkaline phosphatase, and gamma-glutamyl transferase and uLV. 

**Figure 4:**
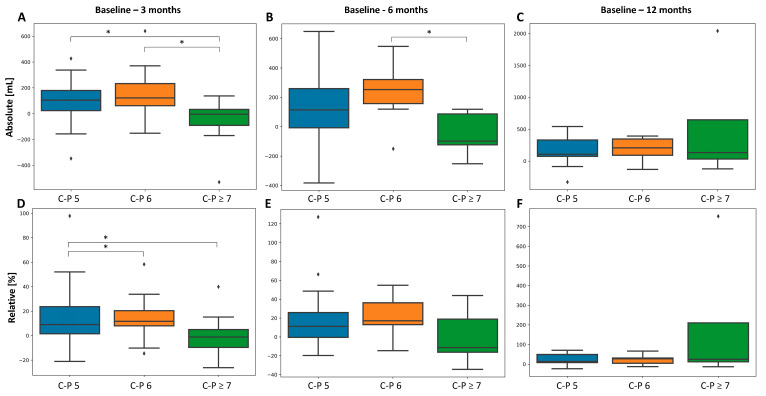
Evolution of uLV according to the baseline Child–Pugh score in absolute (**A**–**C**) and relative (**D**–**F**) values. * *p* < 0.05.

In Child–Pugh A5 or A6, absolute uLV was greater when compared to ≥B7 patients at 3 months (A5, *p* = 0.004, A6, *p* = 0.007, respectively) and 6 months (A5, *p* = 0.072, A6, *p* = 0.031, respectively) vs. baseline. Similar results were obtained at 3 months for relative values but not at later time points. The Child–Pugh score inversely correlated with absolute and relative uLV increase at 3 months (rho = −0.21, *p* = 0.047, Table 3; rho = −0.229, *p* = 0.048, Table 4, respectively); this failed to remain significant in multivariate analysis. Figure 4 is a graphical representation of uLV evolution according to the Child–Pugh score. 

Interestingly, if the Child–Pugh class remained stable at 3 or 6 months post-SIRT (vs. baseline), uLV increased significantly at all time points vs. baseline (all, *p* ≤ 0.015). However, uLV did not increase significantly (3, 6, and 12 months vs. baseline, all *p* > 0.05) when the Child–Pugh class decreased at 3 or 6 months post-SIRT (vs. baseline). Similar results were obtained with the Child–Pugh score.

None of the baseline lab values correlated with treated liver volume evolution. Treated liver volumes decreased significantly regardless of the liver function at all and between time points (all, *p* < 0.05).

#### 3.3.3. Dosimetric Parameters 

Administered ^90^Y activity correlated significantly with absolute uLV increase at 3 months in univariate (rho = 0.28, *p* = 0.01) and multivariate (*p* = 0.003) analyses, but not later on (Table 3). Relative uLV hypertrophy was also impacted by administered ^90^Y activity at 3 and 6 months in univariate analysis (rho = 0.373, *p* = 0.001 and rho = 0.34, *p* = 0.028, respectively, Figure S1), whereas in multivariate analysis, this remained significant at 3 months (*p* = 0.003, Table 4).

Non-tumoral liver activity/dose demonstrated some significant correlations with absolute/relative uLV hypertrophy in univariate analysis but failed in multivariate analysis (Table 3 and Table 4).

Non-tumoral liver activity correlated with absolute treated liver volume decrease at 3, 6, and 12 months (rho = −0.288, *p* = 0.018; rho = −0.371, p = 0.024; rho = −0.664, *p* = 0.001, respectively) and with relative treated liver volume decrease at 12 months (rho = −0.481, *p* = 0.032). None of the other dosimetric parameters impacted the treated liver, tumor burden volume, or laboratory parameters’ evolution at any time point.

#### 3.3.4. Glass versus Resin Microspheres

Absolute uLV hypertrophy was significantly higher at 3 months with glass-microspheres vs. resin when compared to baseline (median, 97 mL (−348–640) vs. 0 mL (−531–371), *p* = 0.029, respectively)). Similar results were obtained at 6 (*p* = 0.014) and 12 months (*p* = 0.027).

Patients treated with glass-microspheres had higher absolute uLV hypertrophy at 3 and 6 months (vs. baseline; *p* = 0.043 and *p* = 0.011, respectively, Table 3) in univariate analysis when compared to resin microspheres. In multivariate analysis, this did not remain significant at 3 months (*p* = 0.215), but a clear trend was observed at 6 months (*p* = 0.058, Table 3). This difference was not observed with relative values (Table 4). Treated liver, spleen, and tumor volumes evolution were similar over time between glass and resin microspheres (all *p* > 0.05), except at 3 months where tumor volume decrease was greater in patients treated with resin vs. glass microspheres (absolute: median, −27 vs. 0 mL, *p* = 0.006; relative: median, −0.418 vs. 0, *p* = 0.018); but not at later time points. Administered ^90^Y activity and tumor and liver activities/absorbed doses were significantly higher with glass vs. resin microspheres (Table S4).

#### 3.3.5. Amount of Treated Liver

The percentage of the treated liver did not impact the absolute uLV increase but did significantly influence relative uLV increase at 3, 6, and 12 months in univariate and, importantly, multivariate analysis (all *p* ≤ 0.01, Table 4). It also negatively correlated with absolute treated liver volume decrease at 3, 6, and 12 months (rho = −0.346, *p* = 0.01, rho = −0.565, *p* < 0.001 and rho = −0.822, *p* < 0.001, respectively). 

The amount of treated liver positively correlated with spleen volume increase at later time points (rho = 0.123, p = 0.263; rho = 0.278, *p* = 0.065; rho = 0.465, *p* = 0.025 at 3, 6, and 12 months, respectively).

A significant correlation was found between the amount of treated liver and total bilirubin increase (rho = 0.311; *p* = 0.006), aspartate transaminase increase (rho = 0.268; *p* = 0.019), and platelet count decrease (rho = −0.296; *p* = 0.01) at 3 months (vs. baseline). Moreover, the magnitude of the treated liver also correlated with PT and albumin decrease (all *p* ≤ 0.042) and Child–Pugh score increase (all *p* < 0.001) at 3 and 6 months post-SIRT. No significant correlation was found with the other lab parameters. The percentage of the treated liver was higher in patients in whom the Child–Pugh class worsened at 3 and 6 months post-SIRT (37.1 ± 24.9% vs. 57.5 ± 33.3%; *p* = 0.006, and 36.6 ± 24.8% vs. 60.8 ± 29.6%; *p* = 0.005, respectively) vs. baseline values. Similar results were obtained for the Child–Pugh score.

#### 3.3.6. Portal Vein Thrombosis

Patients with or without PVT received similar doses/activities (all *p* ≥ 0.11). The presence of PVT did not influence the uLV post-SIRT (Table 3 and Table 4). However, PVT impacted the treated liver with greater hypotrophy observed at 12 months when compared to earlier time points (baseline (*p* = 0.098), 3 months (*p* = 0.045) and 6 months (*p* = 0.015) vs. 12 months, respectively). Partial versus complete thrombosis and PVT extent did not significantly affect volume evolution for both untreated and treated livers. 

#### 3.3.7. Tumor and Spleen

Baseline, increase, or decrease in tumor volume over time did not influence liver volume evolution (treated and untreated, Table 3 and Table 4). Smaller baseline spleen volume influenced relative uLV increase at 3 months in univariate and multivariate analyses (rho = −0.284; *p* = 0.012 and *p* = 0.032, respectively, Table 4). However, no correlation was found with absolute uLV increase or treated liver volume decrease. Other results are presented in Text S3.

## 4. Discussion

SIRT is particularly appealing in the bridge-to-resection scenario. There is a clinical need to better understand volumetric changes in the liver following SIRT with different levels of treatment selectivity, i.e., beyond lobar treatment, which has been the focus of published literature, to better reflect clinical practice.

This is the first report of the 3D quantitative changes in the liver following SIRT in HCC patients using an anatomical, surgically validated, volumetric segment-based approach. This method allowed us to precisely delineate liver segments and investigate volumetric changes post-SIRT based on the actual vascular anatomy and delivered treatment selectivity. A segmental approach may better capture the inhomogeneous ^90^Y microspheres distribution within the treated volume and may prove to be more accurate than the manual drawing of regions of interest in every defined section thickness in the axial plan of cross-sectional imaging, the most used technique [1,3,4,5,6,7,8,9,10,16]. 

We showed that untreated liver kept growing over time, whereas whole liver treatment hampered this regenerative ability. This may be expected, but it is now clearly demonstrated by our results. Even though, in clinical practice, whole liver treatment would not be performed in a neoadjuvant setting, we wanted to assess liver volume evolution in this setting as well. The growth of untreated liver was maximal during the first 6 months post-SIRT (as previously shown with (extended-)lobar SIRT [5,9]) and was still significant between 3 and 6 months (absolute values) in the whole cohort. When looking at extended/lobar treatment, uLV increase was still significant between 6 and 12 months post-SIRT, consistent with previous reports [5]. These data are relevant for surgical candidates, in particular for transplantation, as the waiting time is longer. Although the kinetics of liver hypertrophy are slower when compared to portal vein embolization, SIRT allows for efficient local tumor control and tests tumor biology aggressiveness over time. 

We thoroughly investigated factors influencing untreated liver hypertrophy. We unequivocally demonstrated that the regenerative capacity of the untreated liver decreases with increasing age in the setting of SIRT. A previous report found a negative correlation between uLV increase/month and age [6]. The decline in liver regenerative capacity is a multifactorial age-associated alteration [25]. Although SIRT stimulates liver regeneration, the underlying molecular basis is largely unknown [26].

We showed that the baseline synthetic function of the liver (albumin, PT, and platelets) and bilirubin influenced uLV evolution early post-SIRT (at 3 months). Moreover, we demonstrated that untreated liver hypertrophy was greater with Child–Pugh A (vs. ≥B7) at 3 and 6 months post-SIRT, whereas the Child–Pugh score inversely correlated with hypertrophy at 3 months. Scarce and contradictory data exist in the literature. Like our results, the hypertrophy rate was significantly higher in HCC patients treated using right lobar SIRT with platelet count ≥100/nL and with a low Child–Pugh score [6], whereas no association of platelet count, PT, and total bilirubin with hypertrophy could be observed in other works, which could be explained by heterogeneous cohorts of liver tumors [4,9]. In unilobar HCC patients, Child–Pugh A5 (vs. A6 + B7) was significantly associated with liver hypertrophy [7]. In contrast, the Child–Pugh score did not impact untreated liver hypertrophy in another study investigating lobar SIRT in a mixed cohort of liver tumors [3].

We then investigated how the evolution of liver function post-SIRT influences hypertrophy. Importantly, we showed that uLV did not increase significantly over time when the Child–Pugh class (or score) decreased at 3 or 6 months (vs. baseline) post-SIRT. In the setting of neoadjuvant SIRT, these data could be used to help better triage patients to surgical resection by testing the regenerative capacity of the liver, on top of the biologic test-of-time, while evaluating tumor response to therapy.

We found that higher administered ^90^Y activity was associated with greater untreated liver hypertrophy early post-SIRT regardless of treatment selectivity. No dosimetric parameters related to the non-tumoral treated liver or tumor were predictors of hypertrophy after multivariate analysis. Scarce data exist in the literature. Previous works failed to show a correlation between untreated liver hypertrophy and dosimetric variables (injected activity [2,13], dose to the treated liver [2,3,4]). Palard et al. showed for the first time after lobar SIRT with glass-microspheres in HCC that non-tumoral liver dose correlated with the occurrence of maximal hypertrophy used as a dichotomized variable with the threshold ≥ 10% [7]. In a cohort of primary and secondary liver tumors treated with resin microspheres and in patients with a baseline FLR < 30%, the fraction of the non-tumoral liver dose exposed to ≥30 Gy was the most significant predictor of untreated liver hypertrophy [9]. However, this was not the case when baseline FLR was ≥30%. In these patients, the baseline volume of non-treated liver and injected ^90^Y activity were predictive of hypertrophy. Although the cohort heterogeneity with mixed cancers makes any comparison difficult with our study, taken together, these results highlight the importance of dosimetry planification for optimal liver regeneration.

Glass and resin microspheres achieve similar outcomes in HCC [27]. However, the impact of microsphere type and role of the embolic load (i.e., microsphere number) in liver regeneration setting is not elucidated. We showed for the first time significantly more absolute untreated liver hypertrophy in patients treated with glass-microspheres at 3 and 6 months post-SIRT (*p* = 0.043 and *p* = 0.011) vs. resin-microspheres on univariate analysis, with a clear trend at 6 months on multivariate analysis (*p* = 0.058). These results may, in part, be explained by the significantly higher administered activities and absorbed doses obtained by glass vs. resin microspheres. Interestingly, tumor volume decrease was more pronounced with resin vs. glass microspheres at 3 months post-SIRT. It can be hypothesized that the higher embolic effect of resin microspheres could have contributed to this faster tumor shrinkage. Further research is required to investigate the influence of microsphere type, distribution, and number on tissue volume evolution following SIRT.

Our segment-based approach allowed us to study the influence of treatment proportionality. It negatively impacted liver function at 3 months post-SIRT. Moreover, the percentage of the treated liver was higher in patients in whom the Child–Pugh class/score worsened following SIRT. Importantly, we demonstrated that the magnitude of the treated liver had a clear impact on relative untreated liver hypertrophy at all time points in both uni/multivariate analyses. Thus, it can be hypothesized from our data that in candidates for liver resection and well-preserved liver function, SIRT could be performed in a more non-selective manner to favor contralateral hypertrophy. Moreover, besides the importance of the percentage of treated liver, as shown by our study, the dose received by the perfused volume is key. Grisanti et al. showed that when ≥49% of the non-tumoral liver received at least 30 Gy, the FLR increased to ≥40% [9].

The impact of SIRT on spleen volume and portal hypertension was reported in lobar and whole liver treatments [6,14]. This was confirmed by our study. Moreover, our results add the notion of therapy proportionality to the advent of spleen volume increase. Indeed, no spleen volume increase was observed with more selective treatments, emphasizing the impact of treated volume on liver injury with subsequent fibrosis and tissue remodeling.

In the setting of right radiation lobectomy, PVT was associated with greater untreated liver hypertrophy, and lobar PVT was the only predictor of FLR hypertrophy (≥40%) [3]. In our study, the presence of PVT, its extent (including subgroup analysis with main and/or lobar PVT), and type did not influence untreated liver. Further evidence is needed.

There are limitations in this study. First, it is a retrospective design. Second, treatment subgroups were relatively small due to strict inclusion criteria aiming to limit missing data and increase the robustness of our results. Our sample size was, however, the largest to date [1,2,3,4,5,6,7,8,9], and the variety of subgroups reflects current clinical practice. Third, both glass and resin microspheres were included in the analysis. However, both products were dosed with the partition model, and no difference was shown [27]. Moreover, this reflects clinical practice. Furthermore, dosimetric objectives have evolved over time, and personalized dosimetry is a recent paradigm. Fourth, our cohort was mainly composed of unresectable HCC, and SIRT was not administered to induce FLR hypertrophy but to treat the tumor. Future studies should investigate hypertrophy factors in resectable patients with dedicated SIRT strategies [28].

## 5. Conclusions

Liver function (preserved baseline and stable post-SIRT) favors untreated liver hypertrophy. Younger patients, smaller baseline spleen volume, higher administered ^90^Y activity, and a larger amount of treated liver were associated with a higher degree of untreated liver hypertrophy. These factors should be considered in surgical candidates undergoing neoadjuvant SIRT. Larger prospective studies should be designed to confirm our results.

## Figures and Tables

**Table 1:** Baseline patient and treatment characteristics.

Characteristic	Number of Patients (%)
**Sex** Male Female	76 (86) 12 (14)
**Age**	67 (median, range 17–87)
**Cirrhosis** Present Absent	81 (92) 7 (8)
**Cirrhosis Etiology** Alcohol consumption Hepatitis C virus (HCV) Hepatitis B virus Non-alcoholic steatohepatitis Alcohol consumption + HCV Miscellaneous Unknown	33 (38) 13 (15) 3 (3) 7 (8) 12 (14) 16 (18) 4 (4)
**HCC diagnosis** Biopsy Imaging	44 (50) 44 (50)
**Portal vein thrombosis** Present Main portal Right/left portal Segmental or subsegmental Absent	42 (48) 8 (9) 13 (15) 21 (24) 46 (52)
**ECOG Performance Status** 0 1 2	76 (86) 10 (11) 2 (2)
**Child-Pugh class** A B	74 (84) 14 (16)
**Tumor burden** Unilobar Bilobar Solitary Multiple	59 (67) 29 (33) 51 (58) 37 (42)
**Number of tumors** 1 2 ≥3	41 (46) 20 (23) 27 (31)
**SIRT type** Whole liver Right/left liver Right/left lobe Anterior/posterior sector Segmental/subsegmental	11 (13) 38 (43) 11(13) 6 (6) 22 (25)
**^90^Y-microspheres**	
Glass	57 (65)
Resin Unknown	30 (34) 1 (1)
**Administered activity per patient (GBq)**	
Mean Median	1.9 (95% CI, 1.7–2.1) 1.5 (range, 0.2–3.5)
**Activity to tumor (GBq)** Mean Median	1 (95% CI, 0.9–1.1) 1 (range, 0.2–3.1)
**Dose to tumor (Gy)** Mean Median	329.8 (95% CI, 291.2–368.3) 308.6 (range, 93.5–903)
**Activity to liver (GBq)** Mean Median	0.9 (95% CI, 0.8–1) 1 (range, 0.2–1.7)
**Dose to liver (Gy)** Mean Median	98.3 (95% CI, 71.7–125) 50.3 (range, 7.7–597.7)

**Table 2:** Evolution of absolute volumes.

SIRT		Absolute Median Volume in [mL—Range]				*p*-Value				
		Baseline	3 Months	6 Months	12 Months	Baseline–3 Months	Baseline–6 Months	Baseline–12 Months	3–6 Months	6–12 Months
**All (*n* = 88)**	Treated	610 (63–2749)	521 (20–2223)	492 (13–1522)	278 (30–1206)	**<0.001**	**<0.001**	**<0.001**	**<0.001**	**<0.001**
	Untreated	974 (224–2230)	1041 (320–2329)	1082 (227–2857)	1019 (371–2311	**<0.001**	**0.002**	**0.005**	**0.033**	0.733
	Spleen	400 (128–1681)	454 (156–1673)	449 (153–1680)	388 (154–1233)	**<0.001**	**0.006**	**0.046**	0.684	0.498
	Tumor	52 (1–1675)	48 (1–2264)	16 (1–1287)	10 (1–96)	0.199	**0.008**	**<0.001**	**0.002**	**0.03**
Whole liver(*n* = 11)	Treated	1530 (1176–2749)	1474 (986–2223)	1396 (1296–1522)	1206 (1206–1206)	**0.037**	0.125	1	0.125	1
	Spleen	306 (155–1415)	539 (188–1138)	533 (407–711)	627 (627–627)	0.193	0.625	1	0.625	1
	Tumor	229 (17–1675)	114 (1–1508)	10 (3–63)	14 (14–14)	**0.02**	0.25	1	0.655	1
Right/left liver and lobe (*n* = 49)	Treated	788 (246–1756)	623 (233–1627)	533 (196–1507)	395 (172–785)	**<0.001**	**<0.001**	**<0.001**	**<0.001**	**<0.001**
	Untreated	820 (287–1887)	958 (320–2191)	1003 (227–2120)	984 (371–1591)	**<0.001**	**<0.001**	**0.002**	**0.007**	**0.034**
	Spleen	475 (146–1681)	478 (181–1673)	638 (163–1680)	556 (253–1233)	**<0.001**	**0.02**	**0.001**	0.525	0.067
	Tumor	51 (1–1241)	27 (1–2264)	13 (1–1287)	10 (1–47)	0.904	**0.027**	**0.039**	**0.002**	0.297
Others * (*n* = 28)	Treated	247 (63–1164)	214 (20–1073)	189 (13–951)	159 (30–715)	**0.003**	0.243	**0.039**	0.431	0.203
	Untreated	1270 (224–2230)	1346 (379–2329)	1366 (390–2857)	1054 (896–2311)	0.102	0.747	0.734	1	0.25
	Spleen	391 (128–1041)	376 (156–1264)	358 (153–1067)	233 (154–744)	**0.023**	0.256	1	**0.039**	0.312
	Tumor	48 (4–193)	49 (1–244)	38 (4–357)	8 (7–96)	0.848	0.376	**0.016**	0.136	**0.047**

Note: * correspond to sectorial (V–VIII or VI–VII), segmental or subsegmental treatments.

**Table 3:** Analysis of variables associated with absolute uLV increase.

Parameter	Univariate						Multivariate					
All Versus Baseline	3 Months		6 Months		12 Months		3 Months		6 Months		12 Months	
	t	*p*-value	t	*p*-value	t	*p*-value	f	*p*-value	f	*p*-value	f	*p*-value
Sex	0.486	0.628	−0.29	0.773	−0.26	0.8						
ECOG	−0.3	0.761	0.161	0.873	0.484	0.632						
Cirrhosis	0.443	0.659	−0.57	0.571	−1.22	0.231						
^90^Y-type (resin vs. glass)	2.058	0.043 *	2.64	0.011 *	0.156	0.877	1.567	0.215	3.833	**0.058**		
Portal vein thrombosis	−0.1	0.921	0.902	0.371	−1.03	0.313						
	**rho**	***p*-value**	**rho**	***p*-value**	**rho**	***p*-value**	**f**	***p*-value**	**f**	***p*-value**	**f**	***p*-value**
Age	−0.35	<0.001 *	−0.27	0.043 *	−0.22	0.199	6.448	0.013 *	8.404	0.006 *		
Child-Pugh score	−0.21	0.047 *	−0.11	0.416	0.063	0.714	1.337	0.251				
Administered ^90^Y-activity	0.28	0.01 *	0.21	0.135	−0.03	0.88	9.193	0.003 *				
Non-tumoral liver activity	0.167	0.17	0.251	0.105	0.422	0.025 *					0.905	0.351
Non-tumoral liver dose	0.09	0.447	0.068	0.661	0.445	0.016 *					0.179	0.676
Tumor activity	0.096	0.432	0.038	0.811	−0.27	0.174						
Tumor dose	0.178	0.135	0.291	0.058 *	0.025	0.903			0.67	0.418		
Tumor to liver uptake ratio	0.076	0.516	0.156	0.306	−0.34	0.069 *					0.881	0.357
Amount of liver treated (% of total liver)	0.068	0.537	0.081	0.568	−0.02	0.935						
Spleen volume	−0.19	0.086 *	−0.04	0.801	0.064	0.733	1.684	0.198				
Tumor volume	0.035	0.747	0.076	0.59	−0.018	0.321						

Note: * *p*-value < 0.1 for univariate analysis and *p*-value < 0.05 for multivariate analysis considered statistically significant.

**Table 4:** Analysis of variables associated with relative uLV increase.

Parameter.	Univariate						Multivariate					
All Versus Baseline	3 Months		6 Months		12 Months		3 Months		6 Months		12 Months	
	t	*p*-value	t	*p*-value	t	*p*-value	f	*p*-value	f	*p*-value	f	*p*-value
Sex	0.911	0.365	−0.017	0.986	0.291	0.774						
ECOG	−0.016	0.987	0.791	0.434	0.418	0.68						
Cirrhosis	0.394	0.695	−0.524	0.603	-	-						
^90^Y-type (resin vs. glass)	0.005	0.996	0.682	0.499	−1.303	0.207						
Portal vein thrombosis	0.289	0.773	0.432	0.668	−1.769	0.093 *					0.824	0.378
	**rho**	***p*-value**	**rho**	***p*-value**	**rho**	***p*-value**	**f**	***p*-value**	**f**	***p*-value**	**f**	***p*-value**
Age	−0.253	0.028 *	−0.283	0.069 *	−0.277	0.212	1.574	0.214	2.616	0.116		
Child-Pugh score	−0.229	0.048 *	−0.116	0.464	0.052	0.819	1.466	0.23				
Administered ^90^Y-activity	0.373	0.001 *	0.34	0.028 *	0.272	0.22	9.609	0.003 *	2.295	0.14		
Non-tumoral liver activity	0.198	0.126	0.301	0.079 *	0.432	0.057 *			0.116	0.735	0.089	0.769
Non-tumoral liver dose	−0.012	0.922	−0.023	0.893	0.175	0.447						
Tumor activity	0.084	0.52	0.059	0.742	−0.146	0.552						
Tumor dose	0.066	0.602	0.22	0.204	−0.116	0.637						
Tumor to liver uptake ratio	0.055	0.654	0.106	0.534	−0.27	0.237						
Amount of liver treated (% of total liver)	0.487	<0.001 *	0.552	<0.001 *	0.829	<0.001 *	22.35	<0.001 *	14.26	<0.001 *	8.407	0.01 *
Spleen volume	−0.297	0.01 *	−0.153	0.332	0.074	0.744	5.414	0.023 *				
Tumor volume	0.194	0.096 *	0.187	0.237	0.068	0.764	0.007	0.932				

Note: * *p*-value < 0.1 for univariate analysis and *p*-value < 0.05 for multivariate analysis considered statistically significant.

## Data Availability

The datasets used and/or analyzed during the current study are available from the corresponding author upon reasonable request.

## Supplementary Materials

The following supporting information can be downloaded at: https://www.mdpi.com/article/10.3390/cancers16030586/s1, Text S1: Treatment; Text S2: MRI Acquisition; Text S3: Tumor and Spleen Volume; Table S1: 3D quantitative image analysis of treated liver segments evolution; Table S2: 3D quantitative image analysis of untreated liver segments evolution; Table S3: Evolution of relative volumes; Table S4: Dosimetric parameters of glass- vs. resin-microspheres; Figure S1: Linear regression analysis at 3-, 6- and 12-months of untreated liver volume evolution according to administered activity, non-tumoral liver activity and dose.

## Abbreviations

SIRT = selective internal radiation therapy, HCC = hepatocellular carcinoma, uLV = untreated liver volume, FLR = Future liver remnant, PT = prothrombin time, ^99m^Tc-MAA = technetium-99m-macroaggregated albumin, and PVT = portal vein thrombosis.
