# Supplementary material for: Anatomical Quantitative Volumetric Evaluation of Liver Segments in Hepatocellular Carcinoma Patients Treated with Selective Internal Radiation Therapy: Key Parameters Influencing Untreated Liver Hypertrophy
Source: Cancers (Basel). 2024 Jan 30;16(3):586. doi: 10.3390/cancers16030586 (PMC10854872; doi:10.3390/cancers16030586)
Supplement: Supplementary file 1 [file cancers-16-00586-s001.zip › cancers-2795277-supplementary.pdf]

cancers

MDPI

Supplementary Materials: Anatomical Quantitative Volumetric Evaluation of Liver Segments in Hepatocellular Carcinoma Patients Treated with Selective Internal Radiation Therapy: Key Parameters Influencing Untreated Liver Hypertrophy

Raphaël Girardet, Jean-François Knebel, Clarisse Dromain, Naik Vietti Violi, Georgia Tsoumakidou, Nicolas Villard, Alban Denys, Nermin Halkic, Nicolas Demartines, Kosuke Kobayashi, Antonia Digklia, Niklaus Schaefer, John O. Prior, Sarah Boughdad and Rafael Duran

Text S1: Treatment

A simulation angiography allowed embolization of any potential non-target extrahepatic vessels, followed by selective injection of technetium-99m-macroaggregated albumin (99mTc-MAA) into the tumor feeding arteries (depending on tumor location) and treatment planning. Imaging with single emission computed tomography with integrated CT (SPECT/CT) allowed quantification of treatment volume, dosimetry planning and lung shunt fraction. Predictive therapy planning based on partition model defined several compartments: whole liver volume, perfused volume, tumor perfused volume and non-tumoral perfused volume. Perfused volume was defined for each injection position. Healthy non-perfused liver volume was obtained after the global perfused volume was subtracted to the whole liver. To achieve personalized dosimetry, we calculated the required 90Y-microspheres activity to obtain the desired mean absorbed dose (Gy) in each perfused volume. SIRT (TheraSphere; Boston Scientific and SIR-Spheres; Sirtex), was performed 2-4 weeks following the simulation angiography.

Text S2: MRI Acquisition

All MRIs were conducted on 1.5T and 3T devices (Siemens Healthcare GmbH, Erlangen, Germany). Examinations were performed with a surface body array coil. Liver MRI protocol included: a breath-hold axial and coronal T2WI half-Fourier acquisition single-shot turbo spin-echo (T2WI HASTE), a respiration-triggered fat-suppressed turbo spin-echo T2WI, a free breathing fat-suppressed single-shot echoplanar DWI with tridirectional diffusion gradients using b-factors of 50, 400, 800s/mm^2 and a breath-hold axial dynamic T1WI using a fat-suppressed three-dimensional spoiled gradient-echo sequence, volumetric interpolated obtained before contrast material injection. An intravenous injection of 0.1 mmol/kg (0.2 ml/kg) of gadoterate meglumine (Dotarem; Guerbet, Villepinte, France), at a rate of 3ml/second was then performed. Triple arterial phase images were acquired after a delay of 20 seconds with a Caipirinha (controlled aliasins in parallel imaging results in higher acceleration) Dixon TWIST (time-resolved angiography with stochastic trajectories) VIBE volume interpolated breath-hold examination sequence. Portal and late phase followed, at 70 seconds and 3 minutes respectively.

Text S3: Tumor and Spleen Volume

Baseline tumor volume was similar between glass-microspheres vs resin-microspheres treated patients (mean, 93.7 mL vs 140.2 mL, p=0.19). Tumor volume correlated with absolute and relative spleen volumes at 6-months post-SIRT (rho=0.352, p=0.018; rho=0.307; p=0.043). However, absolute tumor volume increase at 6-months positively correlated with spleen volume increase at 3- and 6-months (rho=0.325; p=0.032; rho=0.289, p=0.060 (trend), respectively).

Baseline spleen volume inversely correlated with baseline platelet count (rho=-0.658, p<0.001), albumin (rho=-0.312, p=0.005) and PT (rho=-0.424, p<0.001). It correlated with

baseline total bilirubin (rho=0.365, $p<0.001$) and Child-Pugh score (rho=0.385, $p<0.001$), but not with ASAT, ALAT, ALP, or GGT. No correlation was found between spleen volume evolution and platelet variation over time post-SIRT.

Administered non-tumoral liver activity correlated with spleen volume increase at 3- and 6-months (rho=0.269, p=0.029 and rho=0.398, p=0.016), but not at 12-months. Administered ^{90}Y-activity also impacted spleen volume increase at 3-months (rho=0.221, p=0.049). Whereas late decrease in spleen volume at 12-months was observed with high delivered tumor activity (rho=-0.771, $p<0.001$). No relationship was found with the other dosimetric parameters.

Table S1. 3D quantitative image analysis of treated liver segments evolution.

Liver Segments	Absolute median volume [mL] (range)				*p*-value				
	**Baseline (*n* = 88)**	**3 mo (*n* = 88)**	**6 mo (*n* = 47)**	**12 mo (*n* = 23)**	**Baseline—3 mo**	**Baseline—6 mo**	**Baseline—12 mo**	**3–6 mo**	**6–12 mo**
I	73 (28-169)	61 (20-195)	71 (13-118)	78 (30-109)	0.485	0.11	0.25	0.455	0.625
II	224 (85-402)	189 (57-413)	225 (79-468)	143 (100-227)	0.068	0.791	0.188	0.233	0.625
III	195 (59-1000)	150 (18-787)	151 (86-719)	106 (68-125)	0.061	0.092	0.062	0.11	0.062
IV	210 (59-528)	177 (38-425)	172 (69-320)	150 (47-214)	0.112	0.002	0.125	0.002	0.188
V	133 (27-342)	113 (17-232)	85 (34-258)	66 (27-143)	<0.001	<0.001	<0.001	<0.001	<0.001
VI	174 (66-417)	144 (23-419)	124 (64-384)	85 (17-316)	<0.001	<0.001	0.005	0.001	<0.001
VII	257 (59-455)	185 (63-479)	191 (92-522)	124 (48-273)	0.001	0.001	<0.001	0.028	0.024
VIII	207 (63-399)	162 (67-396)	158 (44-523)	113 (50-310)	0.001	0.001	<0.001	0.042	0.025
	**Relative mean volume [%] (SD)**				***p*-value**				
	**Baseline (*n* = 88)**	**3 mo (*n* = 88)**	**6 mo (*n* = 47)**	**12 mo (*n* = 23)**	**Baseline—3 mo**	**Baseline—6 mo**	**Baseline—12 mo**	**3–6 mo**	**6–12 mo**
I	5 (1)	5 (2)	4 (2)	5 (2)	0.869	0.455	1	0.542	1
II	13 (5)	12 (5)	12 (5)	9 (3)	0.269	0.85	0.125	0.97	0.438
III	13 (7)	11 (7)	11 (7)	6 (2)	0.093	0.204	0.125	0.424	0.062
IV	14 (5)	13 (6)	11 (3)	9 (4)	0.21	0.042	0.188	0.021	0.438
V	9 (3)	8 (3)	7 (3)	5 (3)	<0.001	<0.001	<0.001	0.001	<0.001
VI	13 (5)	11 (5)	10 (5)	8 (6)	0.002	<0.001	0.007	0.004	0.127
VII	16 (4)	14 (5)	13 (5)	10 (5)	0.002	0.005	0.002	0.159	0.175
VIII	12 (3)	11 (4)	10 (2)	9 (4)	0.002	0.002	0.004	0.191	0.068

Table S2. D quantitative image analysis of ***untreated*** liver segments evolution.

Liver Segments	Absolute median volume [mL] (range)				*p*-value				
	**Baseline (*n* = 88)**	**3 mo (*n* = 88)**	**6 mo (*n* = 47)**	**12 mo (*n* = 23)**	**Baseline—3 mo**	**Baseline—6 mo**	**Baseline—12 mo**	**3–6 mo**	**6–12 mo**
I	80 (9-247)	86 (19-290)	107 (17-328)	60 (22-198)	<0.001	0.011	0.117	0.694	0.865
II	221 (57-527)	240 (34-520)	260 (102-521)	257 (91-501)	0.132	0.09	0.027	0.004	0.712
III	206 (21-556)	224 (42-564)	260 (60-674)	257 (42-572)	0.001	0.007	0.002	0.022	0.89
IV	231 (98-587)	247 (88-599)	253 (68-574)	221 (73-2208	0.098	0.225	0.393	0.948	0.678
V	171 (40-290)	184 (66-345)	168 (68-352)	125 (112-252)	0.019	0.453	0.866	0.325	0.641
VI	252 (27-421)	253 (53-493)	230 (72-465)	179 (30-350)	0.126	0.097	0.82	0.927	0.359
VII	292 (129-560)	298 (120-643)	292 (188-620)	278 (171-502)	0.482	0.089	0.765	0.585	0.147
VIII	207 (116-485)	208 (19-478)	215 (117-511)	201 (161-307)	0.158	0.489	0.039	0.33	0.547
	**Relative mean volume [%] (SD)**				***p*-value**				
	**Baseline (*n* = 88)**	**3 mo (*n* = 88)**	**6 mo (*n* = 47)**	**12 mo (*n* = 23)**	**Baseline—3 mo**	**Baseline—6 mo**	**Baseline—12 mo**	**3–6 mo**	**6–12 mo**
I	5 (2)	6 (3)	6 (3)	5 (3)	0.001	0.007	0.145	0.533	0.734

II	14 (5)	16 (5)	16 (6)	18 (8)	0.033	0.005	0.005	<0.001	0.071
III	14 (6)	16 (7)	17 (7)	20 (9)	<0.001	<0.001	<0.001	0.003	0.243
IV	15 (4)	16 (5)	16 (6)	19 (14)	0.029	0.017	0.002	0.043	0.089
V	10 (4)	11 (3)	10 (4)	11 (4)	0.017	0.523	0.844	0.832	0.312
VI	14 (5)	15 (5)	14 (5)	13 (5)	0.049	0.123	0.57	0.648	0.301
VII	19 (5)	21 (6)	20 (5)	22 (3)	0.06	0.137	0.042	0.272	0.831
VIII	14 (3)	14 (4)	14 (3)	15 (2)	0.256	0.277	0.109	0.72	0.078

Table S3. Evolution of relative volumes.

SIRT	Volume region	Relative volume [%] - mean (SD)				*p*-value				
		Baseline	3-months	6-months	12-months	Baseline—3 mo	Baseline—6 mo	Baseline—12 mo	3–6 mo	6–12 mo
All (*n* = 88)	Treated	45 (29)	40 (29)	35 (27)	26 (20)	**<0.001**	**<0.001**	**<0.001**	**<0.001**	**<0.001**
	Untreated	63 (21)	68 (20)	71 (19)	78 (12)	**<0.001**	**<0.001**	**<0.001**	0.095	0.401
	Spleen	-	-	-	-	-	-	-	-	-
	Tumor	10 (18)	11 (24)	5 (13)	2 (2)	0.552	0.176	**0.006**	0.206	**0.031**
Whole liver (*n* = 11)	Treated	100 (0)	100 (0)	100 (0)	100 (0)	-	-	-	-	-
	Spleen	-	-	-	-	-	-	-	-	-
	Tumor	25 (33)	19 (24)	2 (2)	1 (0)	0.185	0.089	-	0.848	-
Right/left liver and lobe (*n* = 49)	Treated	47 (16)	41 (17)	37 (15)	28 (11)	**<0.001**	**<0.001**	**<0.001**	**<0.001**	**<0.001**
	Untreated	53 (16)	59 (17)	63 (15)	72 (11)	**<0.001**	**<0.001**	**<0.001**	**0.005**	**0.038**
	Spleen	-	-	-	-	-	-	-	-	-
	Tumor	9 (17)	13 (30)	6 (16)	1 (1)	0.304	0.102	**0.015**	**0.004**	0.247
Others* (*n* = 28)	Treated	20 (19)	17 (16)	18 (20)	14 (9)	**<0.001**	**0.025**	0.178	0.847	0.213
	Untreated	80 (19)	83 (16)	82 (20)	86 (9)	**<0.001**	**0.025**	0.178	0.865	0.653
	Spleen	-	-	-	-	-	-	-	-	-
	Tumor	4 (4)	4 (4)	5 (7)	2 (3)	0.808	0.71	**0.017**	0.599	0.06

Note.– *Others correspond to sectorial (V–VIII or VI–VII), segmental or subsegmental treatments.

Table S4. Dosimetric parameters of glass- vs. resin-microspheres

Dosimetric parameter	Glass-microspheres		Resin-microspheres		t-test	
	Median	Range	Median	Range	T-value	*p*-value
Administered ^{90}Y activity (GBq)	1.6	0.25-3.5	1.1	0.6-2.5	1.98	0.048
Non-tumoral liver activity (GBq)	0.98	0.17-3	0.7	0.07-1.35	2.07	0.038
Non-tumoral liver dose (Gy)	62.2	7.7-801	39.8	14.8-83.5	3.58	<0.001
Tumor activity (GBq)	0.87	0.03-3	0.41	0.06-1.56	2.9	0.004
Tumor dose (Gy)	274.5	11.1-1267	184.9	95.5-903	2.04	0.042

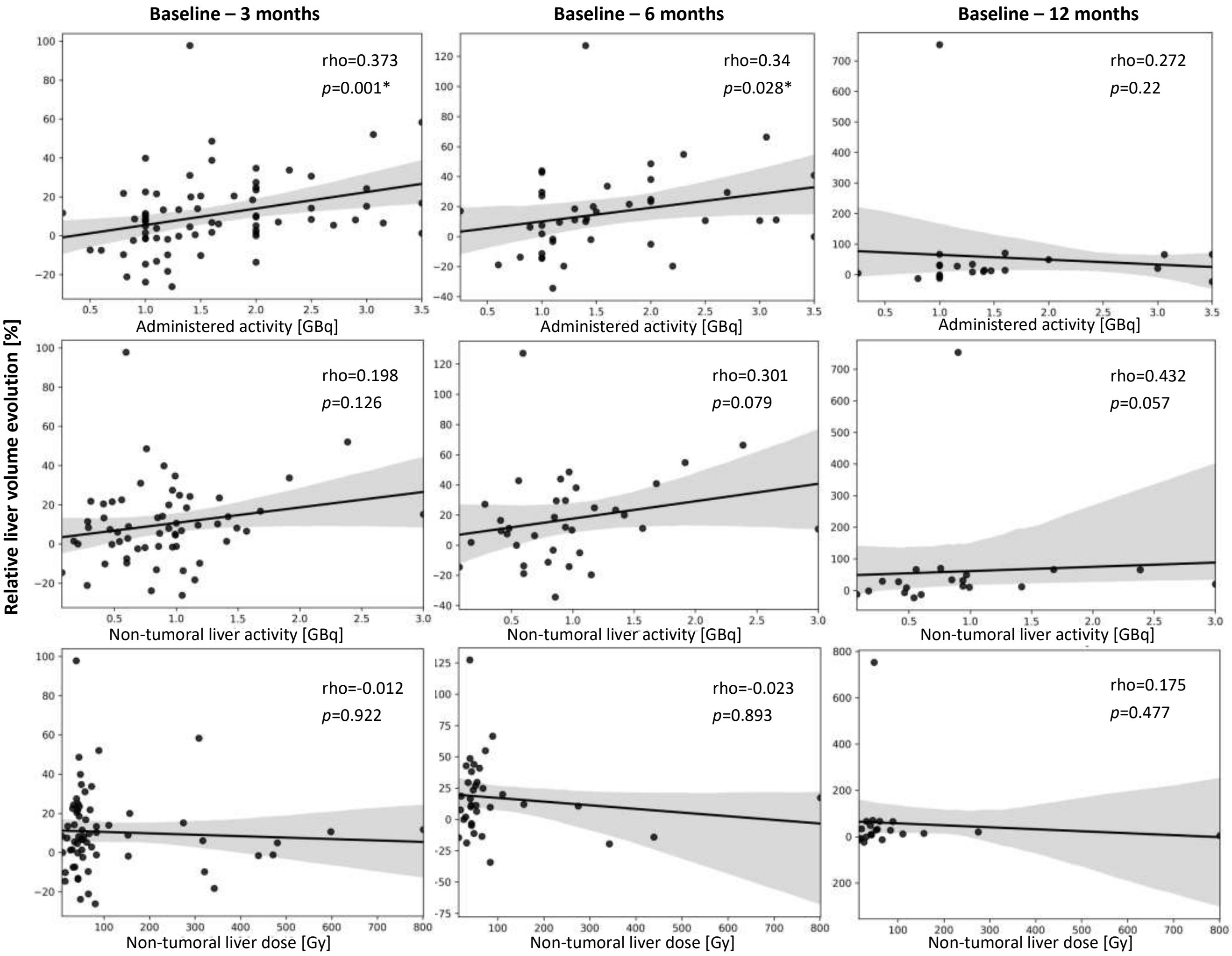

Figure S1. Linear regression analysis at 3-, 6- and 12-months of untreated liver volume evolution according to administered activity, non-tumoral liver activity and dose. * $p < 0.05$.
